# HEALTH-PROMOTING NEIGHBORHOOD RESOURCES ASSOCIATED WITH REDUCED NEURODEGENERATION AND CEREBROVASCULAR DISEASE

**DOI:** 10.1093/geroni/igae098.2124

**Published:** 2024-12-31

**Authors:** Lilah Besser, Pauline Maillard, Elaine Le, Diana Mitsova, James Galvin, Charles DeCarli, Oanh Meyer

**Affiliations:** University of Miami, Boca Raton, Florida, United States; University of California Davis, Davis, California, United States; University of Miami, Coral Gables, Florida, United States; Florida Atlantic University, Boca Raton, Florida, United States; University of Miami, Miami, Florida, United States; University of California Davis, Davis, California, United States; University of California, Davis, Sacramento, California, United States

## Abstract

We aimed to determine if health-promoting neighborhood resources are associated with better brain health measured via structural magnetic resonance imaging (MRI) of older adults. We used data on 195 participants with normal cognition or cognitive impairment from the University of California, Davis Alzheimer’s Disease Research Center. Participants addresses were geocoded to derive neighborhood (Census tract) measures of presence of a library, density of churches, percentage of retail space, and percentage park space. MRI measures included total grey matter volume and white matter hyperintensity volume (WMH), markers of future dementia and cerebrovascular disease risk. Multivariable linear regression that accounted for clustering by Census tract tested associations between the neighborhood characteristics and MRI outcomes, controlling for demographics (e.g., age, race/ethnicity), cognitive status (Clinical Dementia Rating), intracranial volume, population density, and neighborhood socioeconomic status (SES). Models were also stratified by high and low neighborhood SES. Participants were on average 74.9±7.2 years old, had 13.7±4.5 years of education, and 24.1% were Black, 28.9% Hispanic, and 47.1% non-Hispanic White. Individuals in neighborhoods with a library or a higher density of churches had lower WMH volumes, while those in neighborhoods with more churches had greater total grey matter volume. Presence of a library was associated with lower WMH volume for those in low SES neighborhoods, while no associations were observed for those in high SES neighborhoods. Neighborhoods with cognitively stimulating and socially engaging destinations (i.e., libraries and churches) may help reduce cerebrovascular disease and dementia risk by protecting against white matter disease and brain atrophy.

